# Low-molecular-weight metabolites from lactic acid bacteria suppress cervical cancer progression by inhibiting EMT via the Wnt/β-catenin pathway

**DOI:** 10.1515/biol-2025-1264

**Published:** 2026-01-23

**Authors:** Jiawei Xue, Huiying Xiao, Xiaomin Ren, Qifang Yang, Jun Liu

**Affiliations:** Affiliated Fifth Clinical College of Inner Mongolia Medical University, Hohhot, P.R. China; Hohhot First Hospital, Hohhot, P.R. China; Hohhot First Hospital, No.150, South Second Ring Road, Yuquan District, Hohhot, Inner Mongolia 010030, P.R. China

**Keywords:** cervical cancer, lactic acid bacteria, EMT, Wnt/β-catenin pathway

## Abstract

The study aimed to explore the effects of Lactic Acid Bacteria (LAB) supernatant on the epithelial–mesenchymal transition (EMT) of the cervical cancer cell lines HeLa, SiHa, and Hcerepic. We assessed the effect of LAB culture and its supernatant on the proliferation of these cells using the Cell Counting Kit-8 (CCK-8). Scratch assays were performed to evaluate cell migration inhibition. The supernatant was fractionated into >3 kDa and ≤3 kDa components to compare the antiproliferative and antimigratory properties of two fractions. Further, we analyzed the effect of ≤3 kDa supernatant on EMT markers and the Wnt/β-catenin pathway, as well as its effect under LiCl-induced Wnt/β-catenin activation. Grayscale values were obtained using Clinx Chemi Analysis software (ChemiScope 6000, Shanghai, China). LAB and its supernatant significantly inhibited cell proliferation, and the supernatant was more effective. The scratch assay indicated that the LAB supernatant markedly suppressed cell migration. The ≤3 kDa fraction exhibited stronger antiproliferative and antimigratory effects than the >3 kDa fraction. Mechanistically, the ≤3 kDa supernatant reversed EMT by upregulating E-cadherin and downregulating N-cadherin, Vimentin, and Snail. This fraction also inhibited the Wnt/β-catenin pathway, as evidenced by decreased Wnt1, SMAD4, and β-catenin levels, as well as suppressed Gsk-3β phosphorylation. Notably, the ≤3 kDa supernatant maintained its inhibitory effects on proliferation, migration, and EMT even when the Wnt/β-catenin pathway was activated by LiCl. In conclusion, LAB-derived low-molecular-weight metabolites hold therapeutic potential for cervical cancer. The ≤3 kDa supernatant inhibits cancer progression and metastasis by targeting the Wnt/β-catenin pathway and reversing EMT, representing a promising therapeutic approach.

## 1 Introduction

Cervical cancer remains a significant global health challenge, particularly in regions with limited access to preventive measures such as Human Papillomavirus (HPV) vaccination and routine screening [1], [2], [3]. Despite advances in early detection and treatment, it continues to be a leading cause of cancer-related mortality among women worldwide [4], [5], [6]. Cervical cancer cell lines, including HeLa, SiHa, and Hcerepic, play crucial roles in cancer research. HeLa cells, derived from Henrietta Lacks, are highly proliferative and widely used to study cell biology, genetics, and drug screening. SiHa cells, originating from a squamous cell carcinoma, are valuable for investigating HPV and its role in cervical cancer. Hcerepic cells, derived from an HPV-16 positive tumor, provide insights into virus–host interactions and tumorigenesis. These cell lines enable researchers to understand cancer mechanisms, evaluate novel therapies, and advance medical knowledge. Here, we used HeLa, SiHa, and Hcerepic cells for their unique characteristics, including high proliferation rates, HPV positivity, and their capacity to model cancer progression and treatment responses.

One of the key processes underlying cervical cancer progression is the epithelial–mesenchymal transition (EMT), a cellular reprogramming event that enables tumor cells to acquire invasive and migratory properties [7], [8], [9]. During EMT, epithelial cells lose their polarity and cell–cell adhesion markers, such as E-cadherin, while gaining mesenchymal characteristics, including increased expression of N-cadherin, Vimentin, and transcription factors such as Snail [10], [11], [12]. This transition is tightly regulated by signaling pathways, such as Wnt/β-catenin, which plays a central role in promoting EMT and cancer metastasis [13], [14], [15]. Dysregulation of the Wnt/β-catenin pathway is a hallmark of many cancers, including cervical cancer, and is associated with poor prognosis and therapeutic resistance [16], 17].

Lactic acid bacteria (LAB) can inhibit cancer cell proliferation and migration through bioactive metabolites and immune modulation. In addition, LAB can reverse the EMT phenotype by upregulating epithelial markers and downregulating mesenchymal markers, such as N-cadherin, Vimentin, and Snail [18]. The Wnt/β-catenin pathway is a central regulator of EMT and cancer progression. Activation of this pathway promotes EMT by stabilizing β-catenin and driving the transcription of EMT-related genes [19], 20]. Here, we investigated whether the LAB supernatant inhibits EMT by targeting the Wnt/β-catenin pathway, providing novel insights into the anticancer mechanisms of LAB [21], 22].

Natural compounds are being actively evaluated as potential anticancer agents due to their ability to modulate key signaling pathways and inhibit tumor progression with minimal side effects [23]. LAB, commonly used in fermented foods and probiotics, have emerged as a promising source of bioactive metabolites with anticancer properties [24], [25], [26], [27]. LAB-derived supernatants contain several low-molecular-weight compounds, including peptides, organic acids, and exopolysaccharides, which have been shown to exert antiproliferative, antimigratory, and immunomodulatory effects in various cancer models [28], 29]. However, the mechanisms underlying these effects, particularly in cervical cancer, remain poorly understood [29], 30].

In this study, we investigated the anticancer potential of LAB supernatant, focusing on its effects on the proliferation, migration, and EMT of cervical cancer cells. We further examined the low-molecular-weight fraction (≤3 kDa) of LAB supernatant and its role in modulating the Wnt/β-catenin pathway, a key regulator of EMT and cancer progression. Our findings demonstrate that the ≤3 kDa fraction of LAB supernatant markedly inhibits cervical cancer cell proliferation and migration, reverses EMT markers, and suppresses Wnt/β-catenin signaling. These results highlight the therapeutic potential of LAB-derived metabolites as novel agents for cervical cancer treatment and provide new insights into the molecular mechanisms underlying their anticancer effects.

## 2 Methods

### 2.1 Cell culture and treatment

HeLa cells are highly proliferative and immortal, derived from Henrietta Lacks. SiHa cells originate from a squamous cell carcinoma and contain integrated HPV. Hcerepic cells are also HPV-positive, providing insights into viral oncogenesis. Three cervical cancer cell lines, HeLa, SiHa, and Hcerepic, were cultured in Dulbecco’s Modified Eagle Medium supplemented with 10 % fetal bovine serum and 1 % penicillin-streptomycin at 37 °C in a humidified incubator containing 5 % CO_2_.

Inert lactic acid bacteria ATCC55195 were purchased from Beijing Baioubowei Biotechnology Co., Ltd. (Beijing, China) and cultured in de Man, Rogosa, and Sharpe broth (Guangdong HuanKai microbial, China) at 37 °C under anaerobic conditions. The LAB supernatant was collected by centrifugation at 10,000×*g* for 15 min, followed by filtration through a 0.22-µm membrane to remove bacterial cells. Cervical cancer cells were treated with LAB or its supernatant at the indicated concentrations and time points.

### 2.2 Cell proliferation assay

Cell proliferation was assessed using the Cell Counting Kit-8 (CCK-8) assay. Briefly, cells were seeded in 96-well plates at a density of 5 × 10^3^ cells per well and treated with LAB or its supernatant for 24, 48, or 72 h. Next, 10 µL of CCK-8 reagent was added to each well, and the plates were incubated for 2 h at 37 °C. Absorbance was measured at 450 nm using a microplate reader (multiskan go, Thermo scientific). The optimal treatment time was determined to be 48 h.

### 2.3 Cell migration assay

Cell migration was evaluated using a scratch assay. Cells were seeded in 6-well plates and grown to 90 % confluence. A sterile 200-µL pipette tip was used to create a scratch in the cell monolayer. After washing with PBS, cells were treated with 50 μg/mL of > or ≤3 kDa LAB supernatant or its fractions, and images of the scratch were captured at 0 and 48 h using an inverted microscope, and scale bars were added using the LASX software (Leica). The scratch area, or the distance between cells, was analyzed using the ImageJ software. The migration rate was quantified by measuring the change in scratch width over time.

### 2.4 Fractionation of LAB supernatant

The LAB supernatant was fractionated based on molecular weight using ultrafiltration membranes with a 3 kDa cutoff (RC membrane, Millipore Amicon^®^ Ultra, Merck). The supernatant was divided into two fractions (>3 kDa and ≤3 kDa) under a pressure of 2.0 bars. The fractions were lyophilized and reconstituted in PBS for subsequent experiments.

### 2.5 Western blot analysis

Protein expression levels of EMT markers (E-cadherin, N-cadherin, Vimentin, and Snail) and Wnt/β-catenin pathway components (Wnt1, SMAD4, β-catenin, Gsk-3β, and p-Gsk-3β) were analyzed using western blotting. Cells were lysed in RIPA buffer, and protein concentrations were determined using a BCA assay. Equal amounts of protein were separated using SDS-PAGE and transferred to PVDF membranes. Membranes were blocked with 5 % non-fat milk and incubated overnight with primary antibodies at 4 °C, followed by incubation with HRP-conjugated secondary antibodies for 2 h. Protein bands were visualized using an enhanced chemiluminescence (ECL) detection system. The grayscale values were obtained using ChemiScope Capture and ChemiScope analysis software (Clinx Co., Ltd., ChemiScope 6000, Shanghai, China).

### 2.6 Statistical methods

All experiments were performed in triplicate, and data are presented as mean ± standard deviation (SD). Statistical significance was determined using one-way ANOVA followed by Tukey’s post hoc test. A *p*-value of less than 0.05 was considered statistically significant. GraphPad Prism software (v9.5) was used for data analysis and graph generation.

## 3 Results

### 3.1 LAB supernatant inhibits the proliferation and migration of cervical cancer cells

**Figure 1:**
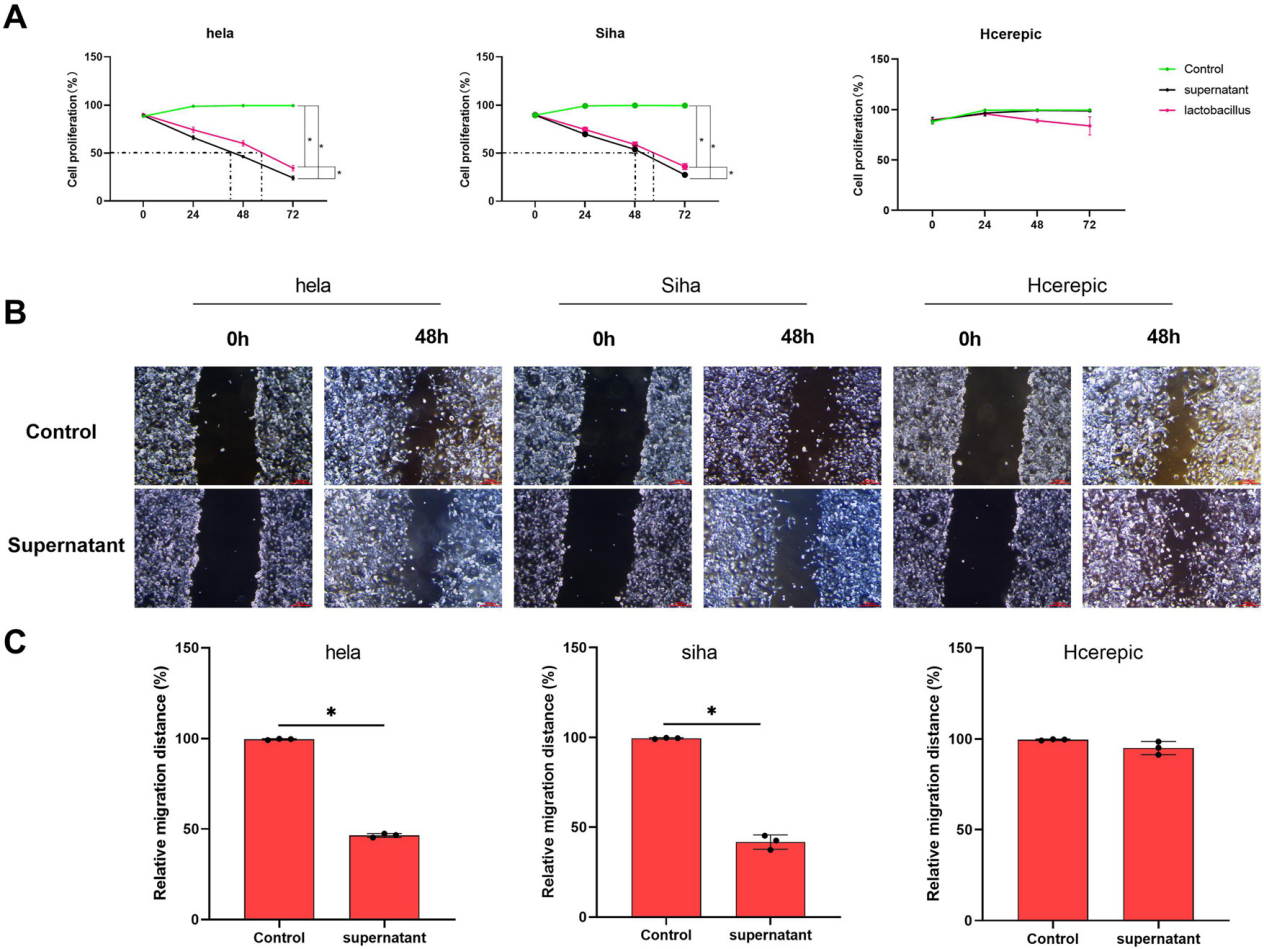
The supernatant of LAB inhibited the proliferation of cervical cancer cells. A. Cell proliferation percentage of cervical cancer cells (Hela, Siha and Hcerepic cell lines) effected by the supernatant of LAB and LAB via CCK-8. B&C. The cell migration ability via the scratch assay. *p<0.05.

We used the CCK-8 assay to measure the proliferation of HeLa cells. Our results demonstrated that both LAB and its supernatant significantly inhibited the proliferation of HeLa cells, and the inhibitory effect of the supernatant was higher than that of LAB alone. Similar results were observed in the SiHa cell line (Figure 1A). The optimal inhibitory effect of the LAB supernatant was observed at 48 h, which was subsequently selected for further experiments. Scratch assays revealed that the LAB supernatant significantly inhibited cell migration in HeLa and SiHa cell lines (Figure 1B and C). However, LAB didn’t inhibit the inhibition or proliferation of the CaSki cell line (Figure 1).

### 3.2 Low-molecular-weight supernatants (≤3 kDa) exhibit enhanced antiproliferative and antimigratory effects

**Figure 2:**
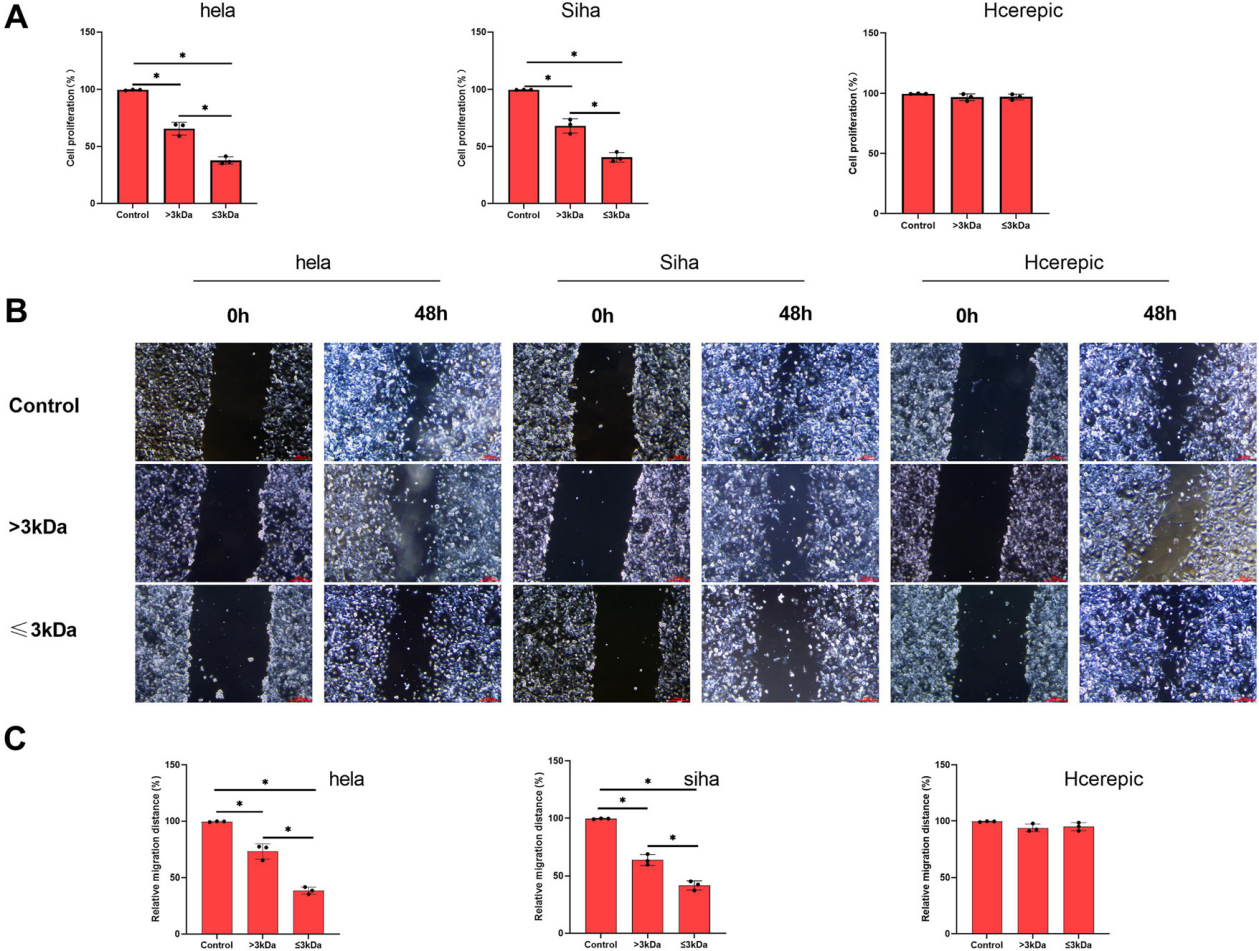
The supernatant of LAB with a molecular weight >3 kD and ≤3 kD inhibited the proliferation of cervical cancer cells. A. Cell proliferation percentage of cervical cancer cells (Hela, Siha and Hcerepic cell lines) via CCK-8. B&C. The cell migration ability via the scratch assay. *p<0.05.

The LAB supernatant contains a heterogeneous mixture of molecules with varying molecular weights. We fractionated the supernatant into >3 kDa and ≤3 kDa fractions to characterize the components with anticancer effects. The ≤3 kDa fraction demonstrated significantly higher inhibition of cell proliferation compared to the >3 kDa fraction in HeLa and SiHa (Figure 2A). In addition, this fraction exhibited superior antimigratory effects in HeLa and SiHa cell lines (Figure 2B and C). However, there was no influence on the normal cell CaSki. These results suggest that low-molecular-weight components (≤3 kDa) of the LAB supernatant are mainly responsible for its potent anticancer activity.

### 3.3 ≤3 kDa supernatant modulates EMT in cervical cancer cells

**Figure 3:**
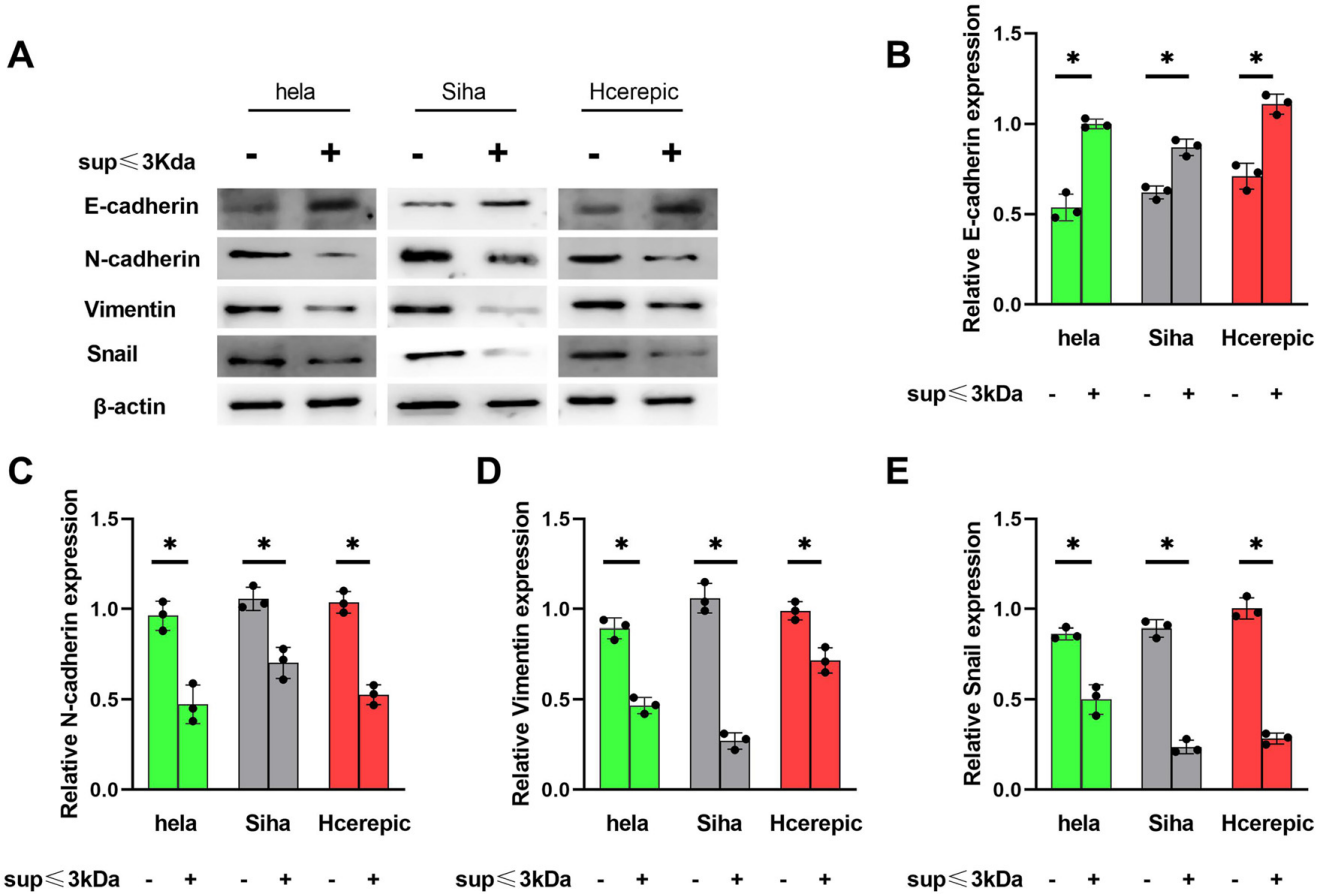
The supernatant of LAB ≤3kD ameliorated the level of EMT. A. E-cadherin, N-cadherin, Vimentin and Snail expression detected by western blot. B–E. Related expression of E-cadherin, N-cadherin, Vimentin and Snail based on the A. *p<0.05.

EMT is a critical process in cancer progression, contributing to increased tumor aggressiveness, migration, invasion, and resistance to apoptosis. We analyzed the expression of key EMT markers, including E-cadherin, N-cadherin, Vimentin, and Snail, to assess the impact of the ≤3 kDa supernatant on EMT. Treatment with the ≤3 kDa supernatant upregulated E-cadherin expression while downregulating N-cadherin, Vimentin, and Snail in HeLa, SiHa, and CaSki cells (Figure 3). These findings indicate that the ≤3 kDa supernatant reverses the EMT phenotype, thereby reducing the aggressiveness of cervical cancer cell lines.

### 3.4 The ≤3 kDa supernatant suppresses EMT via the Wnt/β-catenin pathway

**Figure 4:**
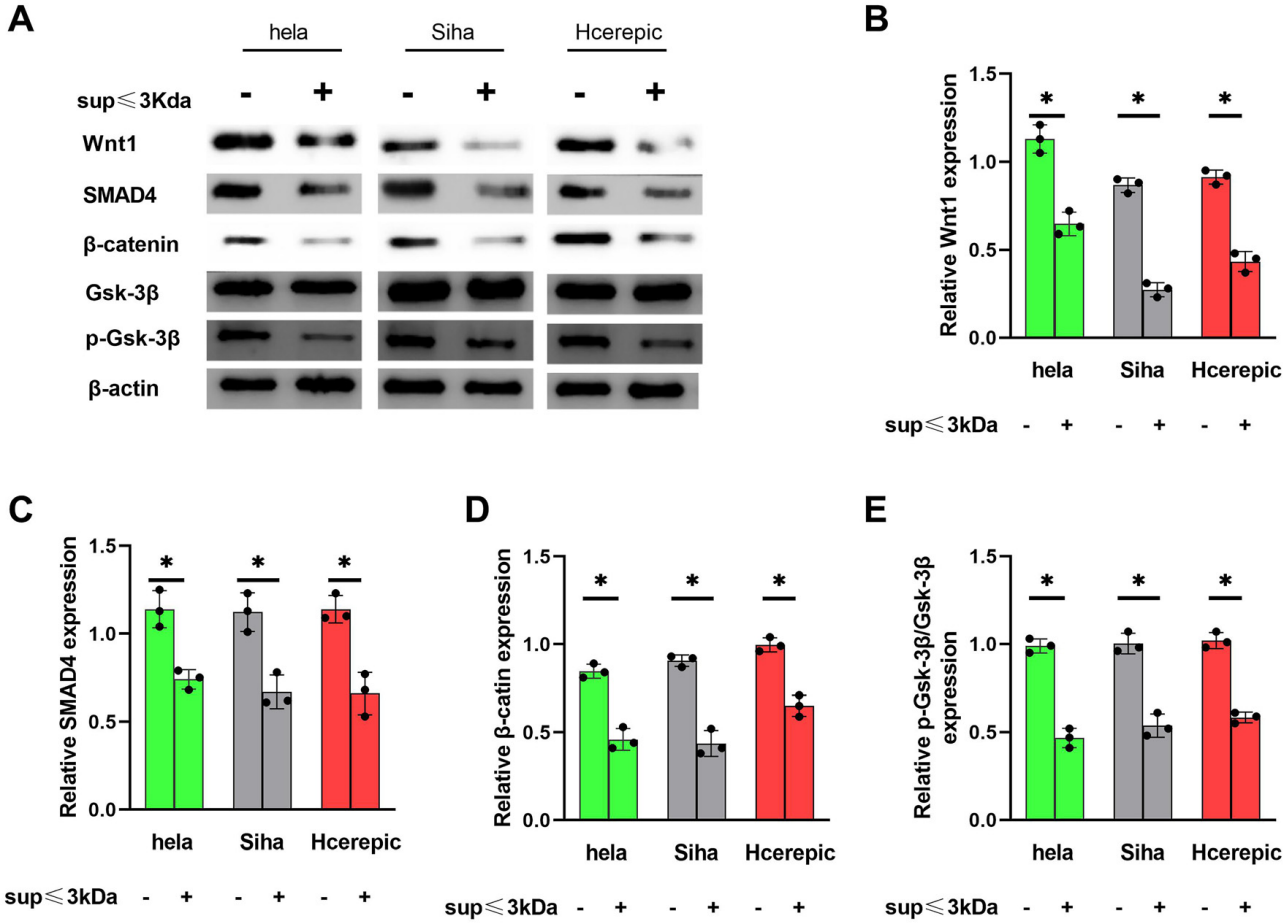
The supernatant of LAB with a molecular weight  ≤3kD ameliorated the level of EMT via Wnt/β-catenin pathway. A. Wnt1, SMAD4, β-catenin, Gsk-3β, p-Gak-3β detected by western blot. B–E. Related expression of Wnt1, SMAD4, β-catenin and p-Gak-3β/ Gak-3β based on the A. *p<0.05.

The Wnt/β-catenin signaling pathway plays a central role in regulating EMT and cancer progression. We examined the expression of key proteins, including Wnt1, SMAD4, β-catenin, Gsk-3β, and p-Gsk-3β, to investigate whether the ≤3 kDa supernatant modulates this pathway. Treatment with the ≤3 kDa supernatant downregulated the expression of Wnt1, SMAD4, and β-catenin while inhibiting the phosphorylation of Gsk-3β in HeLa, SiHa, and CaSki cells (Figure 4). These results demonstrate that the ≤3 kDa supernatant attenuates EMT by inhibiting the Wnt/β-catenin pathway.

### 3.5 The ≤3 kDa supernatant inhibits EMT and cancer cell proliferation even under Wnt/β-catenin pathway activation

**Figure 5:**
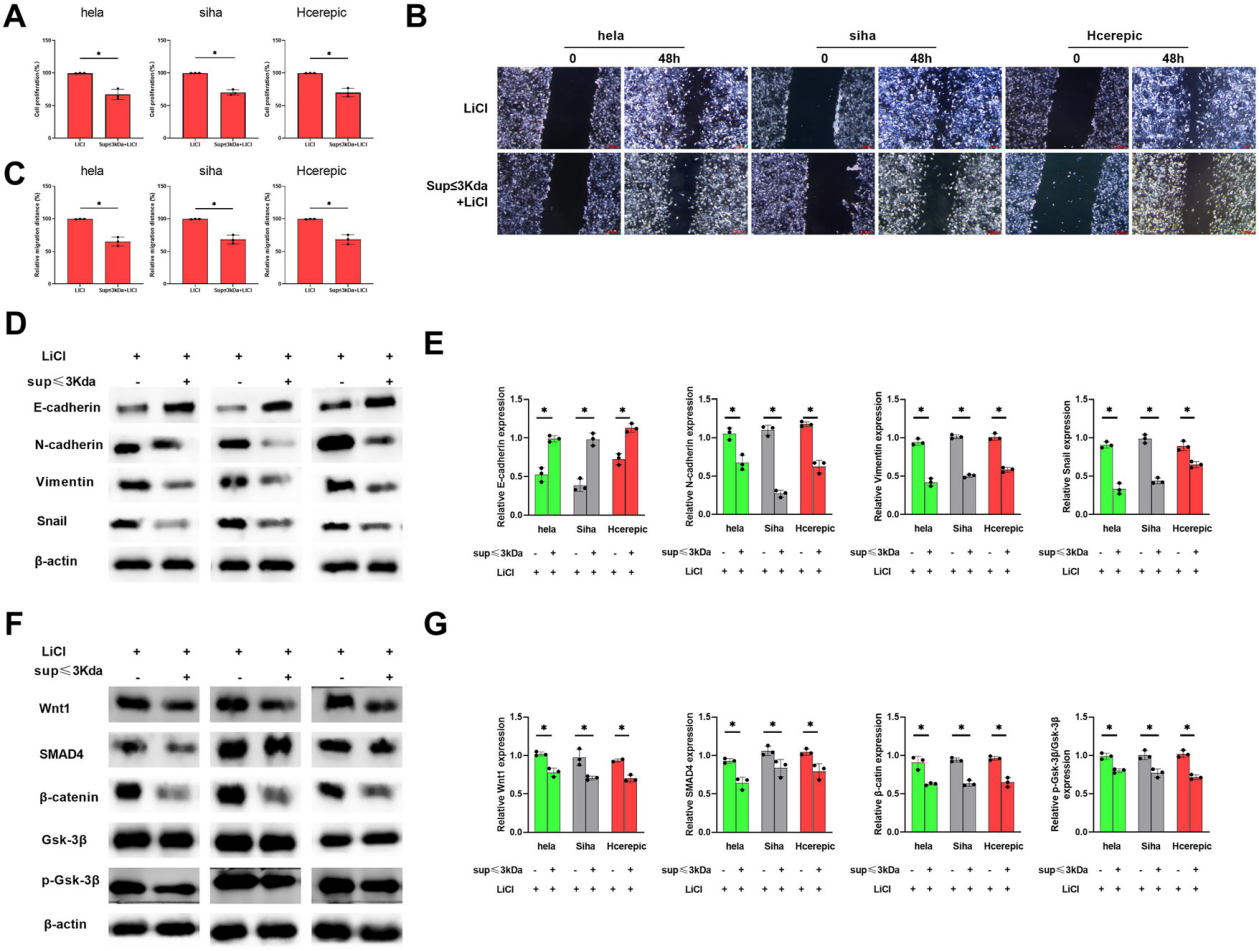
The influence of the supernatant of lactic acid bacteria with a molecular weight  ≤3kD on the the level of EMT when the Wnt/β-catenin pathway was activated by LiCl. A. Cell proliferation percentage of cervical cancer cells. B&C. The cell migration ability via the scratch assay. D&E. Related expression of E-cadherin, N-cadherin, Vimentin and Snail based on the western blot. F&G. Related expression of Wnt1, SMAD4, β-catenin and p-Gak-3β/ Gak-3β based on the western blot. *p<0.05.

We activated the Wnt/β-catenin pathway using LiCl (10 mM, Figure S1) and evaluated the effects of the ≤3 kDa supernatant on this pathway. Despite pathway activation, the ≤3 kDa supernatant significantly inhibited cell proliferation (Figure 5A) and migration (Figure 5B and C) in all three cell lines. Additionally, the supernatant upregulated E-cadherin and downregulated N-cadherin, Vimentin, and Snail (Figure 5D and E). Furthermore, the expression of Wnt1, SMAD4, and β-catenin was decreased, and the phosphorylation of Gsk-3β was inhibited (Figure 5F and G). These findings confirm that the ≤3 kDa supernatant suppresses EMT and cancer progression by targeting the Wnt/β-catenin pathway, even under conditions of pathway activation.

## 4 Discussion

We found that the supernatant of LAB exerts significant inhibitory effects on the proliferation and migration of cervical cancer cells, particularly its ≤3 kDa fraction. These results align with emerging evidence that LAB-derived metabolites possess potent anticancer properties, potentially by modulating key signaling pathways involved in tumor progression. Our data further reveal that the ≤3 kDa fraction of LAB supernatant suppresses EMT and modulates the Wnt/β-catenin pathway, a critical regulator of cancer cell behavior.

The ability of LAB supernatant to inhibit the proliferation and migration cervical cancer cells underscores its potential as a therapeutic agent. Notably, the ≤3 kDa fraction showed more pronounced inhibitory effects compared to the >3 kDa fraction. This finding suggested low-molecular-weight metabolites, such as peptides, organic acids, or other bioactive compounds, may be the primary drivers of bioactivity. Moreover, these observations are consistent with previous studies highlighting the role of small molecules in mediating the anticancer effects of LAB [31]. The observed inhibition of cell migration, demonstrated by scratch assays, further supports the potential of LAB supernatant in mitigating cancer metastasis, a major challenge in cancer treatment. A research reported that the metabolites of *Lactobacillus iners* can enhance the interleukin-17, p53, tumor necrosis factor, and FoxO signaling pathways [32], and the activation of these pathways plays a critical role in cancer prevention and therapy.

EMT is a pivotal process in cancer progression [33], enabling tumor cells to acquire invasive and migratory properties [33], 34]. Our results indicate that the ≤3 kDa fraction of LAB supernatant significantly ameliorates EMT markers in cervical cancer cells. Specifically, the upregulation of E-cadherin and downregulation of N-cadherin, Vimentin, and Snail suggest a reversal of the mesenchymal phenotype, thereby reducing tumor aggressiveness. This finding is particularly significant, as EMT is closely associated with poor prognosis and resistance to therapy in various cancers.

The Wnt/β-catenin pathway plays a central role in regulating EMT and cancer progression [15], 35]. Maspin induces the downregulation of E-cadherin expression and the upregulation of N-cadherin expression in mesenchymal cells by inhibiting the activation of the ITGB1/FAK signaling pathway, thereby promoting the occurrence of EMT in gastric cancer cells. The EMT marker protein Vimentin can transform epithelial cells from a “spindle shape” to a “fibroblast-like” phenotype, accompanied by increased migratory and invasive potential. Vimentin binds to FOXK1 to induce a decrease in E-cadherin expression and an increase in the expression of the transcription factors Snail, Twist, and ZEB1, thereby promoting the EMT of gastric cancer cells. The EMT-related marker ZEB1 promotes EMT occurrence and gastric cancer cell metastasis by activating the Wnt/β-catenin and TGF-β signaling pathways, which leads to decreased E-cadherin expression and increased Vimentin expression. Our data reveal that the ≤3 kDa fraction of LAB supernatant modulates key components of this pathway, including Wnt1, SMAD4, β-catenin, and Gsk-3β. The downregulation of β-catenin and inhibition of Gsk-3β phosphorylation suggest that LAB supernatant may disrupt Wnt/β-catenin signaling, thereby suppressing EMT and tumor progression. This observation was further supported by the results of experiments with LiCl, a known activator of the Wnt/β-catenin pathway. Even under conditions of pathway activation, the ≤3 kDa fraction retained its ability to inhibit EMT and cancer cell proliferation, highlighting its potential as a therapeutic agent. Therefore, the ≤3 kDa fraction of the LAB supernatant is primarily responsible for the observed anticancer effects. This suggests that low-molecular-weight metabolites, such as organic acids, antimicrobial peptides, and other bioactive small molecules, are likely the key components with anticancer activities [32], 36]. Several authors have reported bioactive compounds of LAB supernatant that inhibit cancer cell growth and modulate cellular signaling pathways [36], 37]. For example, lactic acid can create an acidic microenvironment that inhibits cancer cell proliferation. Antimicrobial peptides, such as bacteriocins, can directly affect cell membranes and induce apoptosis [10], 38], 39].

The ability of LAB supernatant, particularly the ≤3 kDa fraction, to target both EMT and the Wnt/β-catenin pathway offers a promising avenue for cancer therapy. Given the challenges associated with current treatments, such as chemotherapy resistance and off-target effects, LAB-derived metabolites represent a natural and potentially safer alternative. Future studies should focus on identifying the specific bioactive compounds in the ≤3 kDa fraction and elucidating their mechanisms of action. Additionally, *in vivo* studies are warranted to validate these findings and assess the therapeutic potential of LAB supernatant in preclinical models.

## 5 Conclusions

Our study reveals that the ≤3 kDa fraction of LAB supernatant inhibits the proliferation and migration of cervical cancer cells by modulating EMT and the Wnt/β-catenin pathway. These findings enhance our understanding of the anticancer properties of LAB and facilitate the development of novel therapeutic strategies targeting EMT and Wnt/β-catenin signaling in cancer.

## Supplementary Material

Supplementary Material
