# Supplementary figures and images for: Low-molecular-weight metabolites from lactic acid bacteria suppress cervical cancer progression by inhibiting EMT via the Wnt/β-catenin pathway
Source: Open Life Sci. 2026 Jan 23;21(1):20251264. doi: 10.1515/biol-2025-1264 (PMC12917598; doi:10.1515/biol-2025-1264)

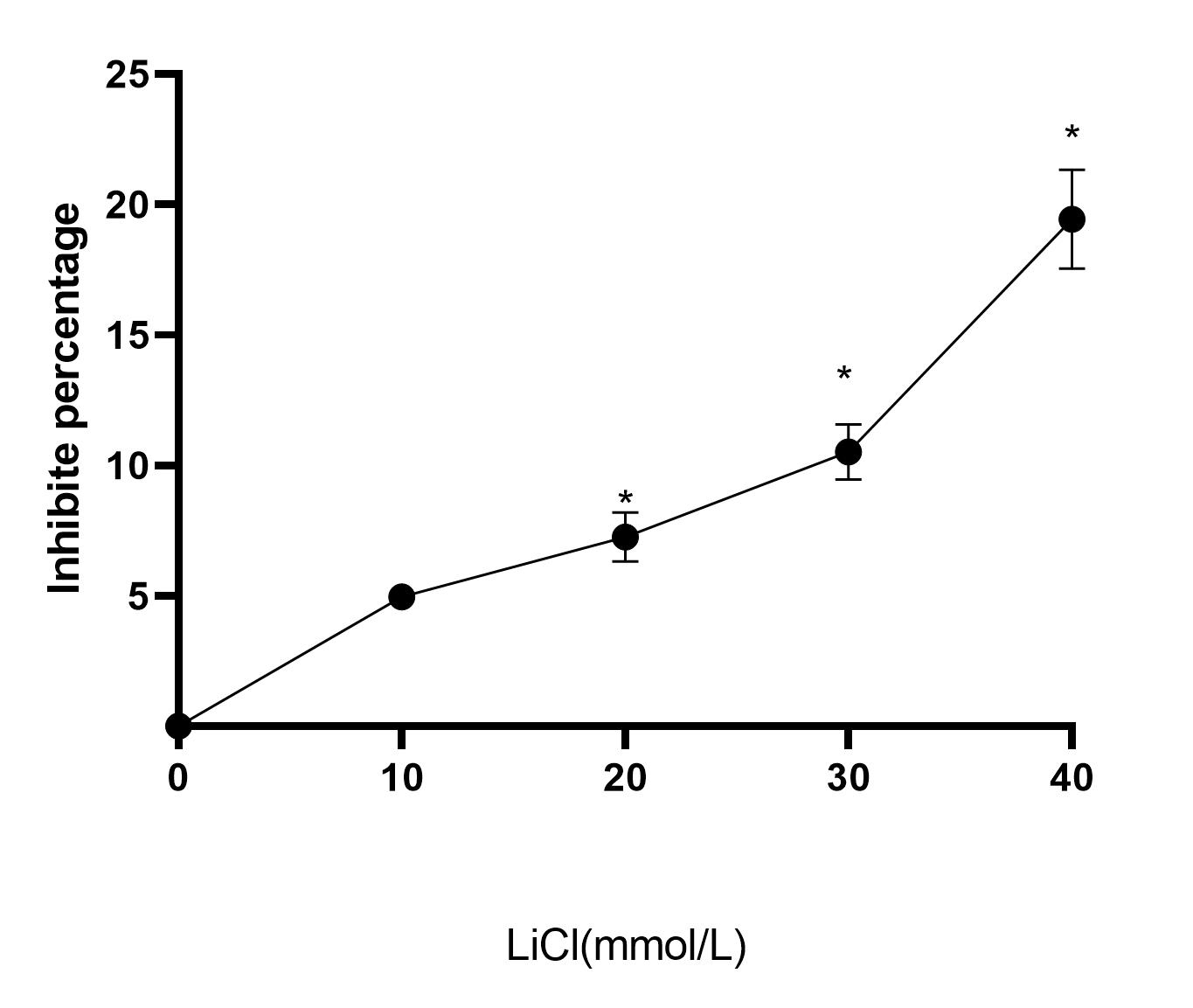

Supplement: Supplementary file 1 — Supplementary Material [file j_biol-2025-1264_suppl_001.jpg]
